# Editorial: Public Health and Primary Care, Is 1+1=1?

**DOI:** 10.3389/ijph.2024.1607347

**Published:** 2024-06-03

**Authors:** Gabriel Gulis, Bernd Rechel

**Affiliations:** ^1^ Unit for Health Promotion Research, University of Southern Denmark, Esbjerg, Denmark; ^2^ Olomouc University Social Health Institute, Palacky University, Olomouc, Czechia; ^3^ European Observatory on Health Systems and Policies, London School of Hygiene and Tropical Medicine, London, United Kingdom

**Keywords:** primary care, public health, collaboration, theme, issue

Over the years 2022–2023 we had the immense pleasure to manage manuscripts submitted to a Special Issue of the International Journal of Public Health addressing examples of primary care and public health collaboration. We launched this Special Issue based on our perception that collaboration between these two vitally important health system elements was lacking. The response we received allows us to say that we were wrong! Within the Special Issue, we managed to accept and publish 46 manuscripts positively evaluated by a range of peer reviewers. All manuscripts are available online at https://www.ssph-journal.org/research-topics/12/public-health-and-primary-care-is-111/articles.

More important than this basic statistic is that the manuscripts covered all major areas where close collaboration between primary care and public health is crucial for promoting health and wellbeing and for preventing diseases, such as:

1. Integration of services: Ensuring seamless coordination between primary care providers (such as family doctors addressing individuals or families at the micro level) and public health agencies (which focus on population health and as such operate at a meso- and macro level) is essential. Early identification of a health issue at the micro level can initiate investigation and action at the meso- and macro levels and through these mechanisms integrated services are likely to lead to better health outcomes and improved cost-effectiveness.
2. Preventive care: Primary care providers play a vital role in preventive care, including vaccinations, screenings, and health education. Collaborating with public health agencies allows for targeted interventions and community-wide health promotion in a manner which allows to tackle social vulnerabilities and ensure more equity.
3. Health equity: Addressing health disparities and promoting equity is a shared responsibility. Collaboration can help identify vulnerable populations, reduce barriers to care, and improve health equity. Identification of vulnerability factors is likely to come from the macro level, but the root causes of disparities are often outside the reach of the traditional health sector, and actors at the meso- or macro levels, acting according to the health-in-all policies approach might be able to address them.
4. Data sharing and surveillance: Public health agencies collect and analyse health data. However, data is in most cases generated at the individual level by a diagnostic procedure often including laboratory samples. Public health agencies summarize and transform the information to the meso-and macro level, making it useable at each level of the health system, as well as for public policy-making outside of it. Sharing this information with primary care providers helps them to make informed decisions, track disease trends, and respond effectively to outbreaks.
5. Community engagement: Collaborating with community organizations and local leaders enhances public health efforts. Primary care providers can engage with community members to understand their needs and tailor services accordingly. They usually possess good knowledge of individuals in the community and, supplemented by the knowledge of public health agencies on decision-makers, stakeholders and processes, creates ideal conditions for community engagement for better health.
6. Emergency preparedness: Public health agencies are usually part of emergency preparedness teams that are responsible for issues related to social and environmental determinants of health and that actively prepare for emergencies (natural disasters, pandemics, etc.). Primary care providers need to be part of these plans to ensure continuity of care during crises.
7. Chronic disease management: Collaboration is also essential for managing chronic conditions (e.g., diabetes or hypertension). Public health agencies and programs need to support primary care in terms of prevention (primary, secondary and tertiary), as well as lead the work on citizen- and patient-oriented health literacy.

In addition to these areas, we were pleased to publish manuscripts discussing new IT technology-based options, such as machine learning, big data and artificial intelligence. It is our strong belief that these new tools can enhance collaboration between public health and primary care. In the very near future, public health agencies should be equipped with both the technological and human capacities to operate AI-based life trajectory and high-risk population identification systems to allow primary care practitioners to better target preventive, diagnostic and treatment regimes. In other words, there is the potential for personalized medicine to meet personalized prevention through AI and other technological tools.

So, are we fully there yet? Despite the success of the Special Issue, caution is required. To reach the goal of 1+1=1 requires embracing a holistic approach to health, as well as shared goals in society. As we observed during the COVID-19 pandemic, this is not always the case. Shared goals require clear definitions, which are related to scientific facts and knowledge, but also to culture, values and social norms. Ultimately, effective collaboration requires open and transparent communication.

